# Effects of Fruit Sizes of Two Camellia Trees on the Larval Sizes of *Curculio styracis* (Roelofs, 1875): Testing the Endoparasitoid Body Size Hypothesis

**DOI:** 10.3390/insects13030246

**Published:** 2022-02-28

**Authors:** Zhiwen Li, Zhongxia Yang, Youzhi Li

**Affiliations:** Hunan Provincial Key Laboratory for Biology and Control of Plant Diseases and Insect Pests, College of Plant Protection, Hunan Agricultural University, Changsha 410128, China; lizhw809718@aliyun.com (Z.L.); zhongxiayang@hunau.edu.cn (Z.Y.)

**Keywords:** body size, *Curculio styracis*, trade-off, adaptive evolution, food constraints

## Abstract

**Simple Summary:**

In endoparasitoids that feed within small discrete resource patches, such as seeds or fruits, body size could be subject to a trade-off: larger size could lead to increased overall fitness but could simultaneously increase the risk of resource depletion and starvation, resulting in a body size just below the host holding capacity. We analyzed the relationship of the larval size of the within-fruits-developing curculionid beetle ***Curculio styracis* (Roelofs, 1875)** and the size of the fruits of its two congeneric host species of Camellia to test this hypothesis. A logistic model can most accurately describe larval size in association with host-fruit size after a series of models were tested. Based on the characteristics of the optimal model, the hypothesis seemed to be confirmed because larvae that developed in host plant with larger fruits had a larger size, and larval size in both host species remained only a little below the host-fruit capacity. The novelty of the study is that this hypothesis is being tested in a more formal way using appropriate mathematical models.

**Abstract:**

The endoparasitoid body size hypothesis suggests that the size of larvae that develop in a single host should be subject to a trade-off: larger size could lead to increase overall fitness but could simultaneously increase the risk of resource depletion and starvation, resulting in a body size just below the host holding capacity. However, this hypothesis has not been rigorously tested using mathematical models thus far. The camellia weevil, *C.* *styracis* (Coleoptera: Curculionidae), is a notorious pest attacking fruits of *Camellia oleifera* Abel. and *C. meiocarpa* Hu., in which the larvae develop within a single fruit and larval development is limited by the available food resources. We developed a feasible method to test this hypothesis. First, five models were used to describe the relationship between larval mass and host size. Then, the minimum fruit threshold that had to be met for ad libitum larval development and the corresponding larval size (*W_a_*) of this threshold were calculated based on the characteristics of the optimal model. Finally, the difference between the measured larval size and the predicted larval size (*W_a_*) was determined. The results showed that (1) the data were better described by a logistic function than any other equation; (2) larval size in both host plants increased with increasing fruit size until leveling off when the fruits were large enough to allow unconstrained larval development; (3) larval size remained just below the host-fruit holding capacity, as there was no difference between the measured and predicted larval sizes (*W_a_*); and (4) larvae developed in host plant with larger fruits had a larger size. These results confirmed the endoparasitoid body size hypothesis.

## 1. Introduction

Insect size, which is closely related to potential fitness and is an important factor driving insect trophic and reproductive strategies, is a key component of life-history evolution and behavioral ecology [1,2,3,4,5,6,7,8,9,10,11,12]. For interacting organisms with antagonistic trophic relationships, such as predation, parasitism, or parasitoid, individual size and body shape adapt to each other [13,14,15,16]. When a prey or host evolves a larger or smaller body size, changes in the body size of its predator, parasite, or parasitoid may follow in step to evolve to maximize fitness, or the predator, parasite, or parasitoid may evolve other strategies [13]. In general, a larger body size is associated with higher fitness, as evaluated based on traits such as survival and potential fecundity [1,7,8,9,10,11,12,17,18,19,20]; thus, evolutionarily, there is underlying pressure to increase body size. However, larger size is not always better; it also comes with potential disadvantages such as a longer development time or higher risk of starvation [7,8,9,10,11,12,21], while smaller individuals benefit from being able to escape the risk of host-food resource depletion and an increased number of suitable hosts [22]. It is reported that body size of organisms can be subject to conflicting selective pressures [7,8,9,10,11,12], especially if these organisms, such as endoparasitoid insects, complete their development within a single host [23,24,25,26]. Under this assumption, the body size of endoparasitoid insects that develop in a single host should be subject to a trade-off between two conflicting selection pressures during long-term adaptive evolution, limiting body size to just below the host holding capacity to avoid the risk of food resource depletion [13,26]. This trade-off is quite generally observed in seed-feeding weevils and other parasitoid insects that develop within a single host [26]. However, few examples have been rigorously tested with appropriate mathematical models.

In this study, we used *C. styracis* as a model to test the endoparasitoid body size hypothesis. The camellia weevil *C. styracis* is a notorious pest attacking fruits of *Camellia oleifera* and *Camellia meiocarpa*. Females oviposit into host fruits and usually lay a single egg per fruit [27,28]. The larvae complete their development within a single fruit, which is prematurely abscised from the tree when the larvae develop to the fourth or fifth instar [29]. Larval size is positively correlated with several fitness variables, such as survival likelihood or potential fecundity [1,8,9,10,11,12,17,18,19,20]. These fitness benefits may have promoted larval size increase. However, it is reported that host fruit size variation is common in the genus *Camellia* [29,30,31], and the small fruits cannot allow larvae inside to increase body size unconstrainedly because of limited resource [32]. Therefore, similar to other seed-feeding weevils [26], the larval size of *C. styracis* could have evolved to a body size that is just below the host holding capacity under conflicting selection pressures (i.e., increased fitness and avoidance of starvation).

We hypothesized that (1) larval size is constrained by food availability and, thus, the size should increase progressively with increasing host fruit size until leveling off at a certain fruit size: in fruits over that size, larval size would no longer be constrained and is predicted to stabilize (i.e., reach the potential larval size); (2) larval size should evolve to remain just below the host holding capacity (i.e., mean larval size should be equal to the larval size predicted by the minimum host threshold necessary to reach the potential larval size); and (3) the larvae developed in *C*. *oleifera*, with larger fruits, should be significantly larger than those developed in *C. meiocarpa*, with smaller fruits.

## 2. Materials and Methods

### 2.1. Study System

Field work was carried out in a *C. oleifera* forest (located at 116 m, 26°17′45.08″ N, 112°26′03.35″ E, in Changning City, Hunan Province, South China) and a *C. meiocarpa* forest (located at 284 m, 26°56′14.93″ N, 111°28′08.32″ E, in Shaoyang County, Hunan Province, South China). The two sites are located approximately 200 kilometers apart, with a hilly landscape. They cover areas of approximately 7 hm^2^ and 6 hm^2^, respectively.

*C. styracis* is a weevil that feeds on camellia fruits [27,33,34]. The reported crop losses of *C. oleifera* caused by this weevil are extremely variable, usually ranging from 22.3 to 60.2% and sometimes from 60 to 100% [27]. *C. meiocarpa* fruits are damaged more seriously than those of *C. oleifera* [31,35]. Weevil females excavate a hole through the fruit coat using their rostrum and then turn around and oviposit inside the fruit. The peak of oviposition occurs from mid-June to mid-July [27]. The weevils usually lay one egg per fruit, and the occurrence of multiple larvae per fruit was extremely rare [27,28]. The larvae go through five instars [36] and complete their development inside a single fruit by feeding on the seeds; when the larvae develop to the 4th~5th instar, the infested fruits are usually prematurely abscised [29]. Larvae of dropped fruits remain inside until their development is finished. Mature larvae make a round hole with a diameter of 3~5 mm before leaving the fruits to overwinter in earthen cells in the soil in the first year, where they pupate from August to November in the next year. The emerged adults pass over the second winter in the soil and come out during late April and early May of the third year [28]. Once larvae leave fruits, their foraging behavior will stop.

### 2.2. Sampling Methods

#### 2.2.1. Dry Mass of Larvae in Fallen Fruits

In 2018, forty trees in the *C. oleifera* forest were randomly selected for sampling [18,37,38]. This survey was conducted every 10 days from the beginning of fruit drop to the end of fruit harvesting. All fallen fruits under the tree canopy were collected, after which indehiscent fruits with no exit holes were selected, placed individually in plastic cups, and numbered before their linear dimensions (length and width) were measured to the nearest 0.01 mm with a digital caliper. Each fruit was checked regularly at 8:00 and 20:00 every day to register emerged larvae. The newly exited larvae were individually placed in 1.8 mL refrigerated centrifuge tubes and stored at −20 °C. In each set of collected samples, each fruit was dissected two weeks after the emergence of the last larva to check for remaining larvae and to assess whether the available food had been depleted. The whole experiment was carried out at room temperature. Finally, the collected larvae were placed in an oven (Jinghong Experimental Equipment Co., LTD, Shanghai, China) at 60 °C and baked to constant mass, and the dry mass was then measured to the nearest 0.1 mg with an electronic balance (Shimadzu, Shanghai, China).

#### 2.2.2. Dry Mass of Larvae in Sleeve Experiments

Forty trees in the *C. meiocarpa* forest were randomly selected for sampling in our study areas, and 11 branches with fruits were randomly selected from each tree. Then, nylon mesh sleeves (40 × 60 cm, made of 40 mesh nylon) were installed in mid-May 2018 (before females laid eggs) on branches to prevent weevil females from ovipositing their eggs in the fruits inside the sleeves. From 2 June to 21 August (the oviposition season), sleeves were selected using a sampling regime in which one sleeve was randomly selected from each tree every 8 days and removed from the branch to allow females to lay eggs in the fruits for 8 days, after which the sleeve was reinstalled. The fruits inside the sleeves were all collected 30 days after oviposition, when the larvae had almost reached maturity (third to fifth instars) and the infested fruits had ceased to develop and were close to falling off; these fruits were taken to the laboratory for the same treatment described above.

### 2.3. The Model

The fresh mass of camellia fruit cannot be used as a measure of available food resources in weevil-infested fruits because of the consumption of the seeds. Fruit shape varies on these trees, which may produce orange-, spherical-, peach-, umbilical-, and oval-shaped fruit. The seeds inside the fruit are approximately spherical, so the relative size of fruits can be estimated with the following formula:

(1) $$V=\frac{4}{3}\pi {\left(\frac{D}{2}\right)}^{3},$$

where *V* is the adjusted volume of the fruits, and *D* is the diameter of the fruits.

Bonal and Muñoz [26] built an empirical model (a negative exponential model) to assess the relationship between *C. elephas* larval size and *Quercus ilex* acorn size. Previous studies have shown that the relationship between insects and ages approximately follows a sigmoidal curve [39,40,41,42,43], and the Richards, von Bertalanffy, Gompertz, and logistic equations are commonly used to describe the course of mass increases with age [43,44,45,46,47]. In the case of the *Camellia* weevil studied herein, the larvae can develop to different stages (ages) in hosts of different sizes; therefore, we hypothesized that the relationship of larval mass versus host size should conform to a sigmoidal curve.

In this study, the larval size increment per unit increment of host fruit size was defined as the marginal effect (d*W*/d*V*). Four sigmoidal equations and a negative exponential equation [26] were selected to describe the relationship between the larval size of *C. styracis* and host fruit size:

(1) The Richards equation,

(2) $$W=W_{m}{\left(1\pm be^{-KV}\right)}^{-\left(1/n\right)};$$

(2) The von Bertalanffy equation,

(3) $$W=W_{m}{\left(1-\frac{1}{3}e^{-K\left(V-V_{I}\right)}\right)}^{3};$$

(3) The Gompertz equation,

(4) $$W=W_{m}e^{-e^{-K\left(V-V_{I}\right)}};$$

(4) The logistic equation,

(5) $$W=\frac{W_{m}}{1+e^{-K\left(V-V_{I}\right)}};$$

(5) The negative exponential equation,

(6) $$W=W_{m}-e^{-K\left(V-d\right)},$$

where *W* is the larval dry mass, *V* is the volume of the host fruit, *W_m_* is the asymptote mass (i.e., the potential larval size), *K* is the marginal effect constant, *b* is the integration constant, *n* is the shape parameter determining the position of the inflection point of the curve, *V_I_* is the host fruit volume at the inflection point, and *d* is the displacement of the entire function along the *V*-axis in the negative exponential equations.

The accuracy of the models could be determined based on the mean square error (*MSE*) and Akaike’s information criteria (*AIC*). Smaller *AIC* or *MSE* values for any model indicated that a certain model fit the data better than the others [48,49].

### 2.4. Data Analysis

SPSS 13.0 and Origin 9.0 were used for data analysis and mapping. Nonlinear fitting between larval dry mass and fruit size was performed via the Levenberg–Marquardt method. An independent samples *t*-test or an ANCOVA was used to analyze the differences in fruit size or in larval size between *C. oleifera* and *C. meiocarpa*, respectively, and a single-sample *t*-test was used to analyze the significance of the difference in larval size between the measured and predicted values. The correlation of larval dry mass with either the immature (egg and larva) period in host fruit or the number of days needed for larval emergence after the collection of dropped fruit was analyzed using linear regression.

## 3. Results

**Figure 1:**
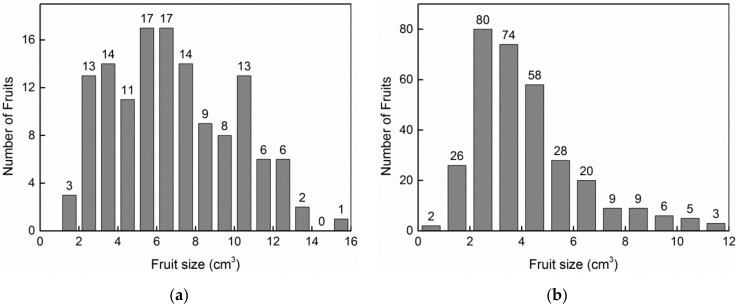
Frequency of the weevil-infested fruits: (**a**) *C*. *oleifera*; (**b**) *C*. *meiocarpa*.

**Figure 2:**
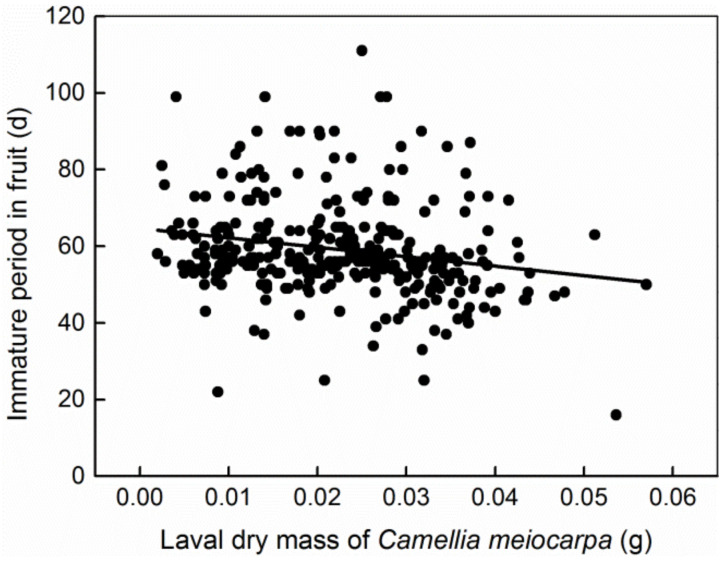
Linear correlation between the immature (eggs and larvae) period (d) in fruit and the larval dry mass (g) of *C*. *meiocarpa*.

**Figure 3:**
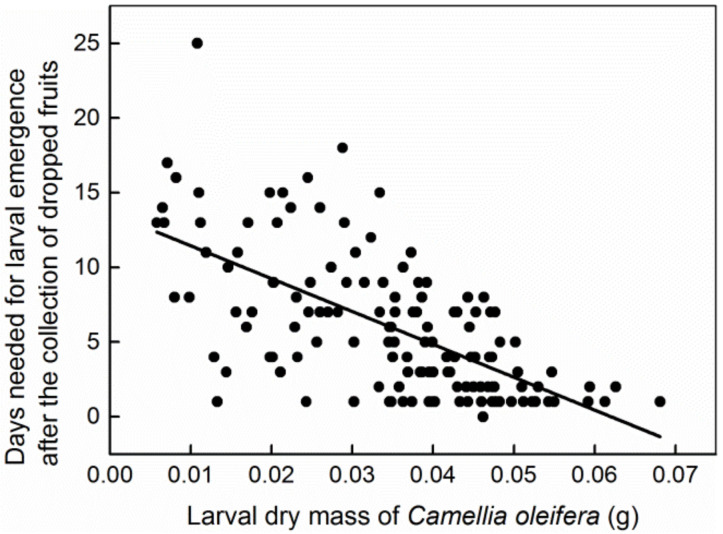
Linear correlation between the days needed for larval emergence after the collection of dropped fruit and the larval dry mass (g) of *C*. *oleifera*.

**Figure 4:**
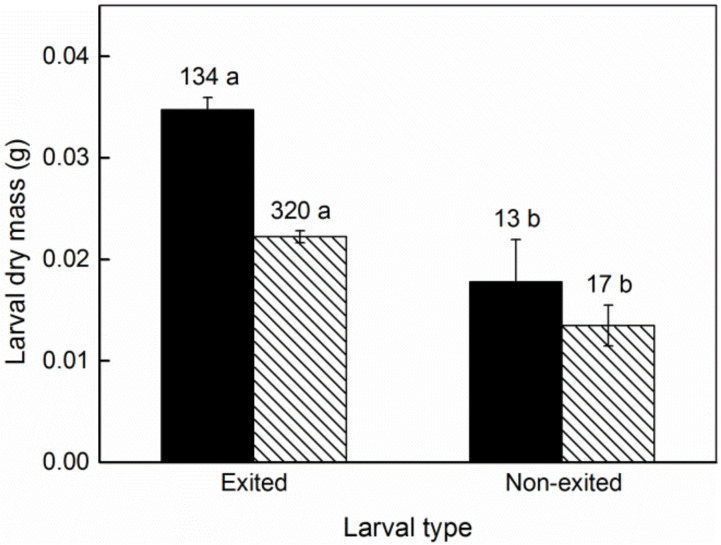
Difference in larval dry mass (mean ± *SE*, g) between exited and non-exited larvae. Black bars, *C*. *oleifera*; lined bars, *C*. *meiocarpa*. Values above the error bars indicate sample sizes. Different letters indicate significant differences in larval dry mass (*t* test, *p* < 0.05).

A small number of fruits produced two larvae in each plot. We did not use the fruits with two larvae due to the small sample size. There were 134 *C. oleifera* fruits (ranging from 1.08 to 15.33 cm^3^) and 320 *C. meiocarpa* fruits (ranging from 0.67 to 11.83 cm^3^) with one larva per fruit, and the size frequency of the infested fruits is shown in Figure 1. The greater the larval mass, the shorter the immature (egg and larva) period in host fruit was, and the fewer days were needed for larval emergence after the collection of dropped fruit (*R*^2^_adj_ = 0.0408, *p* = 0.0002, Figure 2; *R*^2^_adj_ = 0.4082, *p* = 0.0000, Figure 3). Thirteen and 17 larvae failed to emerge from their host fruits of *C. oleifera* and *C. meiocarpa*, respectively, and their dry masses were significantly lower than those of the larvae that emerged normally (*C. oleifera*: *t*_145_ = 4.187, *p* < 0.0001; *C. meiocarpa*: *t*_335_ = 3.336, *p* = 0.0009; Figure 4).

**Figure 5:**
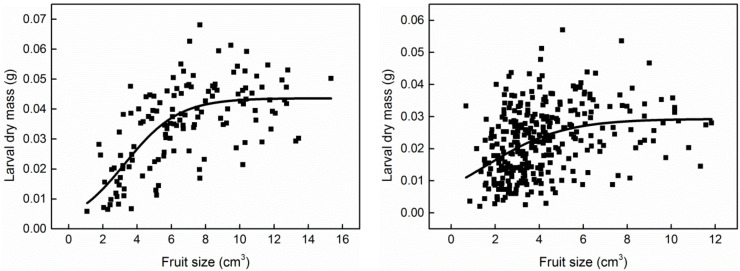
Relationships between larval dry mass (g) and fruit size (cm^3^) based on logistic model: (**a**) *C*. *oleifera*; (**b**) *C*. *meiocarpa*.

The data were suitable for fitting with all five models (*p* < 0.0001, Table 1), and comparisons based on *MSE* and *AIC* showed that four sigmoidal functions led to a better fit to the data than the negative exponential function. The dry masses predicted from these equations were rather similar throughout the middle portions of the curves but deviated from one another at either end. In both hosts, the logistic model showed the lowest *MSE* and *AIC*, indicating that it was the most appropriate function for describing larval mass in association with fruit size (Table 1, Figure 5). Therefore, the logistic curve was taken as an example to analyze the dynamic characteristics of the models in this study.

**Figure 6:**
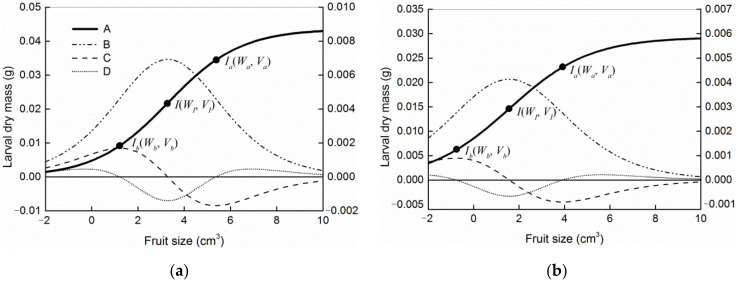
Dynamic characteristics of the marginal effect of *C. styracis* larval development: (**a**) *C*. *oleifera*; (**b**) *C*. *meiocarpa*. A, logistic model; B, marginal effect (d*W*/d*V*); C, first derivative of marginal effect; D, second derivative of marginal effect. *I* (*W_I_*, *V_I_*), the inflexion point of the logistic model; *I_b_* (*W_b_*, *V_b_*), the critical point (before the inflexion point) between the slow change stage and fast change stage of marginal effect; *I_a_* (*W_a_*, *V_a_*), the critical point (after the inflexion point) between the fast change stage and asymptotic change stage of marginal effect.

The logistic curve, marginal effect, and first and second derivatives are shown in Figure 6. The maximum marginal effect occurred when the first derivative was equal to zero. Then, the inflection point of the logistic curve was found to be *I* (*W_I_*, *V_I_*). When the second derivative of the marginal effect was equal to zero, the first derivative reached its maximum or minimum value, indicating that the marginal effect changed most dramatically; the corresponding points (i.e., *I_b_* (*W_b_*, *V_b_*) and *I_a_* (*W_a_*, *V_a_*)) were the two critical points in the logistic curve. Thus, the curve could be divided into three stages based on these points: a slow change stage (before point *I_b_*, *V* < *V_b_*), a fast change stage (between points *I_b_* and *I_a_*, *V_b_* < *V*< *V_a_*), and an asymptotic change stage (after point *I_a_*, *V* ˃ *V_a_*) of the marginal effect. The coordinates of these two points in the Richards, von Bertalanffy, and Gompertz models could be obtained by the same method (Table 1).

**Figure 7:**
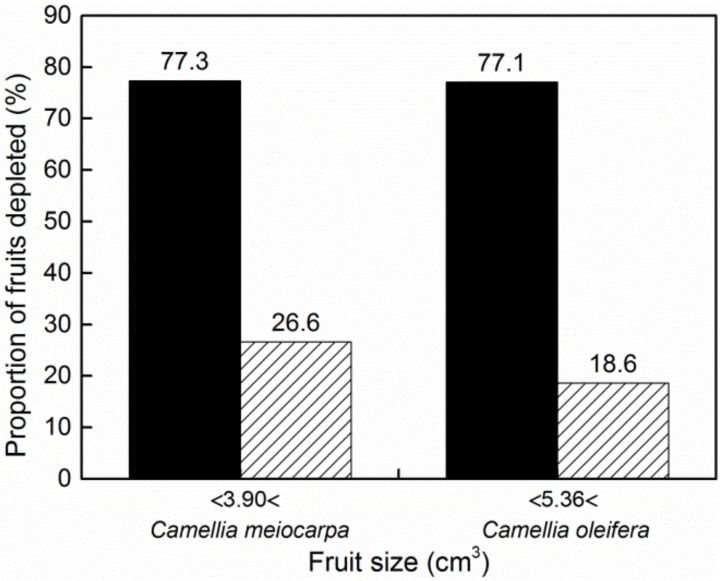
Proportions of fruit types depleted by the larvae smaller (black bars) or larger (lined bars) than *V_a_* value (cm^3^) based on the logistic model in which larval size levels off.

According to the logistic curve, the two critical points may have extremely important biological meaning. There were almost no data distributed in the scatter plots of larval dry mass versus host fruit size (Figure 5) in the slow change stage (*V* < *V_b_*), in which the larvae could develop to 21.13% (*W_b_*/*W_m_* × 100%) of their potential size. This means that the larvae cannot mature in fruits smaller than *V_b_*. For fruits larger than *V_b_*, the frequency of different fruit sizes is shown in Figure 1. The value of *V_b_* calculated with the logistic model presented the minimum fruit threshold for larval emergence. Larval size increased with host fruit size and nearly leveled off at the critical point of *I_a_* (*W_a_*, *V_a_*) (Figure 6a), at which larval size reached 78.87% (*W_a_*/*W_m_* × 100%) of the potential size. Therefore, *W_a_* is a parameter value just below the potential size, and *V_a_* is the minimum fruit threshold that must be reached for ad libitum larval development. Once the fruit size exceeded this threshold (*V_a_*), the proportion of depleted fruits rapidly decreased (Figure 7).

Mean fruit size is an obvious measure of host capacity, and mean dry mass is a measure of larval size at this capacity. There was no significant difference between the mean larval dry mass (see Table 2) and *W_a_* (see Table 1) according to a single-sample *t*-test (*C. oleifera*: *t*_133_ = 0.301, *p* = 0.764; *C. meiocarpa*: *t*_319_ = −1.616, *p* = 0.107, respectively), indicating that the measured larval size was just below the host capacity.

In contrast, each potential larval size (*W_m_*) in *C. oleifera* fruit calculated from the five models was approximately 1.5 times that in *C. meiocarpa* fruit (Table 1), and the ANCOVA also indicated that the larvae showed a larger size (*F*_1,450_ = 30.628, *p* < 0.0001, Table 3) when they developed in the fruits of *C. oleifera*, which produces larger fruits (*t*_452_ = 9.180, *p* < 0.0001, Table 2), presenting a lower risk of resource depletion (χ^2^_1_ = 16.255, *p* < 0.0001).

## 4. Discussion

Little work has focused on the relationship between endoparasitiod insects and their hosts. Our results showed that the logistic model provided the best fit to the data among the tested models, and that host fruit size of *C. styracis* does constrain parasitoid size. This limitation is mainly imposed by the availability of food. In small fruits, the seeds are depleted, and the larvae cannot reach their potential size. There have been many reports about the limitations on endoparasitoid size imposed by host size [5,22,25,37,50], but few quantitative studies have been carried out through modeling to address this issue [26]. Our models estimated the fruit-holding capacity of the two host species by calculating the maximum potential size that the larvae could reach. Moreover, based on the characteristics of the model rather than a conditional algorithm (an empirical method), the model calculated the minimum fruit threshold necessary to reach the maximum potential larval size and the corresponding larval size (*W_a_*) predicted by this threshold. In this way, we can explicitly assess the suitability of a host for larval development according to its size. By testing the difference between mean larval size and *W_a_*, we could further assess whether the larvae have evolved such that their size remains just below the host holding capacity.

In this study, small fruits were often depleted (Figure 7, Table 3), and the curve of the larvae whose dry mass increased with increasing host fruit size conformed to the logistic model, showing that the body size of the weevil larvae increased as much as possible. In general, larval size is a key factor associated with fitness; for example, larger larvae show a higher likelihood of survival over the long overwintering period and will become larger adults with higher potential fecundity [1,5,18,51]. In this study, the mass of non-exited larvae was significantly lower than that of the larvae that emerged normally (Figure 4), indicating that these larvae may be not vigorous enough to exit from fruits due to lack of adequate or high-quality nutrition and thus have longer internal fruit duration, while the larger the larvae were, the shorter their immature period in the fruits was observed to be (Figure 2), and the shorter the time required for larval emergence after fruit collection. Therefore, larger larvae would avoid the risk of predation to a certain extent. We think that these fitness benefits could be one of the main factors responsible for the evolution of *C.* *styracis* body size. As a matter of fact, this evolutionary trend seems to be quite widespread within the Curculio group [26]. Comparative interspecific studies have shown that shifts to exploit larger seeds are followed by a body size increase, provoking morphological diversification between the Curculio species [3]. However, larval size cannot be increased without restriction to avoid the risk of starvation because the amount of resources available to endoparasitoid insects that develop in a single host fruit is limited [22,26]. Our results showed that there was no significant difference between the mean dry mass of the larvae and the values (*W*_2_) predicted by the minimum fruit threshold that had to be met for larval development ad libitum, indicating that the larvae of *C. styracis* have evolved such that their body size remains just below the host-fruit holding capacity under conditions of resource limitation.

More interestingly, we found that larval size differed between the two *Camellia* species at the population level. This phenomenon has also been reported in other *Curculio* species [5]. For endoparasitoids that feed within small discrete resource patches, such as seeds or fruits, body size at the population level was affected not only by resource availability and quality but also by intraspecific competition [26,52,53,54,55]. In our study systems, the occurrence of multiple larvae per fruit was extremely rare. There were three (for *C. oleifera*) and seven (for *C. meiocarpa*) fruits with two larvae in each fruit, accounting for 2.2% and 2.1% of the weevil-infested fruits, respectively. This meant that larval size in this study is mainly determined by resource availability, while larval intraspecific competition has almost little effect on it. Obviously, the fruits in *C. oleifera*, compared with those in *C. meiocarpa*, have a larger size and thus greater holding capacity for larval development. Therefore, a larger size of the within-*C. oleifera*-fruits-developing larvae is expectable if the endoparasitoid body size hypothesis holds.

## 5. Conclusions

A method was proposed to test the endoparasitoid body size hypothesis using *C. styracis* and two congeneric host species of *Camellia* that differ in average fruit size. We tested a series of models and found that a logistic model most accurately describes larval size in association with host-fruit size. Based on the characteristics of the optimal model, the potential larval size was calculated to estimate host-fruit holding capacity of the two host species, and the endoparasitoid body size hypothesis seemed to be confirmed because (1) larval size in both host plants increased with increasing fruit size until leveling off when the fruits were large enough to allow unconstrained larval development; (2) larval size remained only a little below the host-fruit capacity; and (3) larvae developed in host plant with larger fruits had a larger size.

## Figures and Tables

**Table 1:** Fitting effect between larval dry mass (g) and fruit size (cm^3^), model parameters and coordinates of points *I_b_* and *I_a_*. I: Negative exponential model, $W=W_{m}-e^{-K\left(V-d\right)}$; II: Richards model, $W=W_{m}{\left(1\pm be^{-KV}\right)}^{-\left(1/n\right)}$; III: von Bertalanffy model, $W=W_{m}{\left(1-\frac{1}{3}e^{-K\left(V-V_{I}\right)}\right)}^{3}$; IV: Gompertz model, $W=W_{m}e^{-e^{-K\left(V-V_{I}\right)}}$; V: logistic model, $W=\frac{W_{m}}{1+e^{-K\left(V-V_{I}\right)}}$, where *W* is the larval dry mass, *V* is the volume of the host fruit, *W_m_* is the asymptote mass (i.e., the potential larval size), *K* is the marginal effect constant, *b* is the integration constant (host size scale parameter), *n* is the shape parameter determining the position of the inflection point of the curve, *V_I_* is the host fruit volume at the inflection point, and *d* is the displacement of the entire function along the *V*-axis in the negative exponential equation. *I_b_* and *I_a_* are the two critical points (before and after the inflexion point, respectively) of the models at which the second derivative of the marginal effect was equal to zero and the first derivative reached its maximum and minimum values, respectively.

Host	Model	Goodness-of-Fit			Parameters of Models				Coordinates of *I_b_* and *I_a_*	
		*MSE*	*AIC*	*p*	*W*_m_	*K*	*V_I_*/*d*	*n*	*I_b_* (*V_b_*, *W_b_*)	*I_a_* (*V_a_*, *W_a_*)
*Camellia* *oleifera*	I	1.063 × 10^−4^	−1222.99	<0.0001	0.0459	0.3077	−9.0492			
	II	1.050 × 10^−4^	−1223.71	<0.0001	0.0434	0.7423	26.5858	1.7188	(1.6644, 0.0123)	(5.7147, 0.0359)
	III	1.050 × 10^−4^	−1224.63	<0.0001	0.0446	0.4281	2.1216		(0.2635, 0.0008)	(3.9796, 0.0273)
	IV	1.047 × 10^−4^	−1225.02	<0.0001	0.0442	0.4825	2.4970		(0.5025, 0.0032)	(4.4915, 0.0302)
	V	1.043 × 10^−4^	−1225.61	<0.0001	0.0436	0.6356	3.2885		(1.2165, 0.0092)	(5.3606, 0.0344)
*Camellia* *meiocarpa*	I	9.760 × 10^−5^	−2952.09	<0.0001	0.0306	0.3145	−11.5828			
	II	9.722 × 10^−5^	−2952.37	<0.0001	0.0284	1.6766	5615.5633	10.0592	(2.2433, 0.0175)	(5.3014, 0.0268)
	III	9.741 × 10^−5^	−2952.71	<0.0001	0.0300	0.4013	0.4625		(−1.5196, 0.0005)	(2.4446, 0.0184)
	IV	9.735 × 10^−5^	−2952.92	<0.0001	0.0297	0.4433	0.8100		(−1.3612, 0.0022)	(2.9812, 0.0203)
	V	9.716 × 10^−5^	−2953.54	<0.0001	0.0293	0.5649	1.5676		(−0.7638, 0.0062)	(3.8989, 0.0231)

**Table 2:** Comparisons of measurements performed on infested fruits between the two *Camellia* species. The mean is presented as the mean ± *SE*.

Measurements	*Camellia oleifera*	*Camellia meiocarpa*	Independent Samples *t*-Test or Pearson Chi-Square Test	
Fruit size (cm^3^)	6.87 ± 0.27	4.14 ± 0.12	*t*_452_ = 9.180	*p* < 0.0001
Larval dry mass (g)	0.0348 ± 0.0012	0.0222 ± 0.0006		
Percentage of fruits depleted	35.8%	56.6%	*χ*^2^_1_ = 16.255	*p* < 0.0001

**Table 3:** Analyses of covariance of larval dry mass of two host species of *C. styracis* subjected to different fruit size.

Source of Variation	Larval Dry Mass(g)		
	*d*.*f*.	*F*	*p*
Model	3	86.552	<0.0001
Host species (H)	1	30.628	<0.0001
Fruit size (F)	1	116.591	<0.0001
H × F	1	4.530	0.034
Error	450		

## Data Availability

The data presented in this study are available in the article.
